# Oral Lactulose vs. Polyethylene Glycol for Bowel Preparation in Colonoscopy: A Randomized Controlled Study

**DOI:** 10.7759/cureus.14363

**Published:** 2021-04-08

**Authors:** Jagdeep Jagdeep, Gaurish Sawant, Pawan Lal, Lovenish Bains

**Affiliations:** 1 Department of Surgery, Maulana Azad Medical College, New Delhi, IND

**Keywords:** colonoscopy, lactulose, polyethylene glycol, palatability, serum electrolytes

## Abstract

Background

Colonoscopy is the method of choice to evaluate colonic mucosa and the distal ileum, allowing the diagnosis and treatment of many diseases. Appropriate bowel preparation necessitates the use of laxative medications, preferentially by oral administration. These include polyethylene glycol (PEG), sodium picosulfate, and sodium phosphate (NaP). Lactulose, a semi-synthetic derivative of lactose, undergoes fermentation, acidifying the gut environment, stimulates intestinal motility, and increases osmotic pressure within the lumen of the colon.

Methods

In this prospective randomized controlled study, we analyzed 40 patients who presented with symptomatic bleeding per rectum and underwent bowel preparation either with lactulose or polyethylene glycol for colonoscopy. The quality of bowel preparation and other variables like palatability, discomfort, and electrolyte levels were analyzed.

Results

The majority of the patients (90%) were comfortable with the taste of lactulose solution, whereas the PEG group patients (55%) were equally divided on its palatability. On lactulose consumption, 40% of patients reported nausea/vomiting and around 10% of patients complained of abdominal discomfort. Serum sodium levels showed insignificant changes from 4.33 ± 0.07 mEq/L to 4.21 ± 0.18 mEq/L while potassium also remained similar from 4.26 ± 0.03 mEq/L to 4.22 ± 0.17 mEq/L. The mean Boston Bowel Preparation Score (BBPS) in patients who received lactulose solution was 6.25 ± 0.786 and in those who received PEG solution, it was 6.35 ± 0.813 (P-value = 0.59).

Conclusions

Lactulose is a significantly more palatable form of bowel preparation and causes minor discomfort. It has a good bowel cleansing action comparable to PEG without causing any hemodynamic changes. It can be considered a cheaper and safe alternative for bowel preparation in colonoscopy in low-resource settings.

## Introduction

Colonoscopy is an investigation for the evaluation of colonic mucosa [1]. Nowadays, a flexible colonoscope is used most commonly [1]. The indications include investigation for colorectal cancer screening especially those with a family history, intestinal bleeding, iron deficiency anemia, chronic inflammatory diseases of the colon, changes in bowel habits, and diarrhea of unexplained origin. The success of colonoscopy depends on many factors but colonic cleaning is the key factor [2]. The quality of bowel preparation aims to empty the colon of all fecal material, permitting adequate visualization of the mucosal surface. The preparation of the colon is considered an appropriate factor directly associated with the correct diagnosis, lower chances of complications, and patient complaints [3].

Polyethylene glycol is a non-absorbable electrolytic solution. It has been shown to be nontoxic but requires a large volume to be consumed [4-5]. Lactulose is a disaccharide, semi-synthetic derivative of lactose. Lactulose is readily available in the market and can be consumed directly, as it has a likable taste profile, there is no need for reconstitution, and it is cost-effective and well-tolerated by patients. Though both are readily available, there is a paucity of literature about lactulose usage for colonoscopy in the Indian context. This study aims to compare the efficacy of polyethylene glycol and lactulose in colonoscopy preparations.

## Materials and methods

This study was designed as a randomized, controlled, single-center blinded study conducted at Maulana Azad Medical College, New Delhi. Formal prior approval was taken from the institutional ethical committee. In our study, patients with age >18 years and bleeding per rectum were included while patients with ileostomy, prior colonic resection, or having bowel obstruction, and pregnant patients were excluded. All patients underwent a standard clinical and laboratory evaluation that includes routine blood investigations like hemogram, total leukocyte counts, platelet count, coagulation profile, blood sugar (fasting/postprandial), blood urea, serum creatinine, and per rectal examination. Serum electrolytes were sent before bowel preparation was started and on the morning of the procedure (at 8 am).

**Figure 1:**
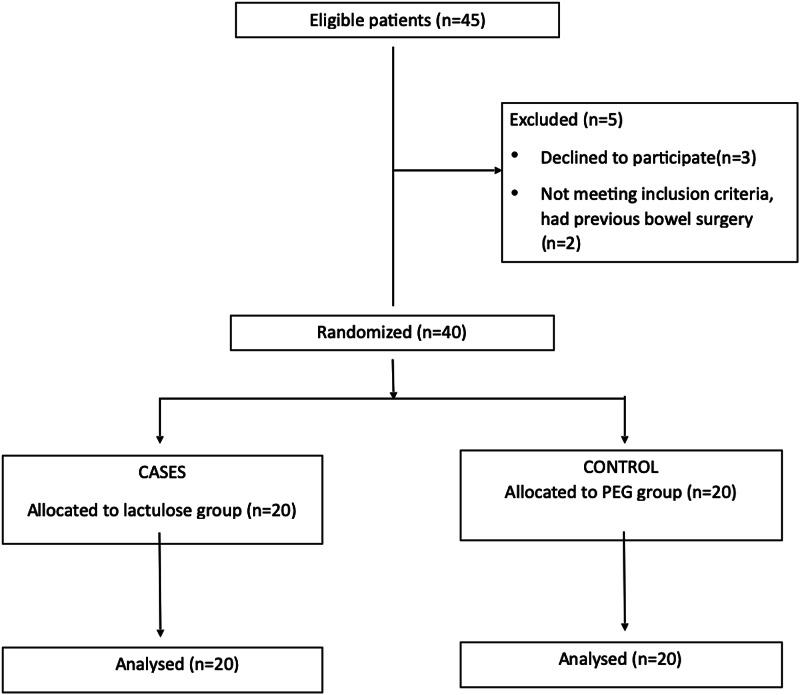
CONSORT diagram and allocation CONSORT: Consolidated Standards of Reporting Trials

A sample size of 40 was calculated having a confidence interval of 95% and an alpha of 5%. Randomization was done by computer-generated random numbers on the day of the procedure. The endoscopist was blinded from the study. The patients were randomized into two groups; in one group of patients, bowel preparation was done using lactulose solution (cases), and in the other group, by using polyethylene glycol solution (control), both of which were in the hospital supply (Figure 1). One group was given to drink one packet of polyethylene glycol (137.15 g) (brand: PEGLEC, GR Medex, Nagpur, Maharashtra, India) with 1 liter of water while the other group was given 300 milliliters of lactulose (10 gm/15 ml) (brand: Duphalac, Actiza Pharmaceutical, Surat, Gujarat, India) with 700 milliliters of water, from 5-7 pm on the night prior to colonoscopy. Both groups were monitored for any side effects and were asked about nausea, vomiting, and abdominal discomfort on the morning of the study. The patients were also asked if they found the solution palatable or not. The colonoscopy was done by the same consultant for all patients; findings during the procedure and the quality of the preparation were recorded with the Boston Bowel Preparation Scale (BBPS) based on visual estimation of fecal residues observed during the examination (Table 1) [6]. After the procedure, patients were observed in the recovery room. The patient was assessed again in the evening. If there was any discomfort, the patient was observed overnight and vitals monitored. Once the patient was deemed fit, he was discharged with symptomatic treatment.

**Table 1:** Boston Bowel Preparation Scale (BBPS) score

SCORE	MUCOSA
0	Mucosa not visible
1	Portion of the mucosa is visible
2	Minor residue but mucosa is seen well
3	Entire mucosa is seen well with no residue

Statistical analysis

The collected data were entered in Microsoft Excel (Microsoft Corporation, Redmond, WA) and then was analyzed and evaluated using the Statistical Package for the Social Sciences (SPSS) software, version 24.0 (IBM Corp. Armonk, NY). The student t-test was used for statistical analysis. The data were analyzed by Fisher's exact test for uniformity in patient distribution according to gender. P-value less than 0.5 was considered significant at a 95% confidence level; the power of our study was 80%.

## Results

**Figure 2:**
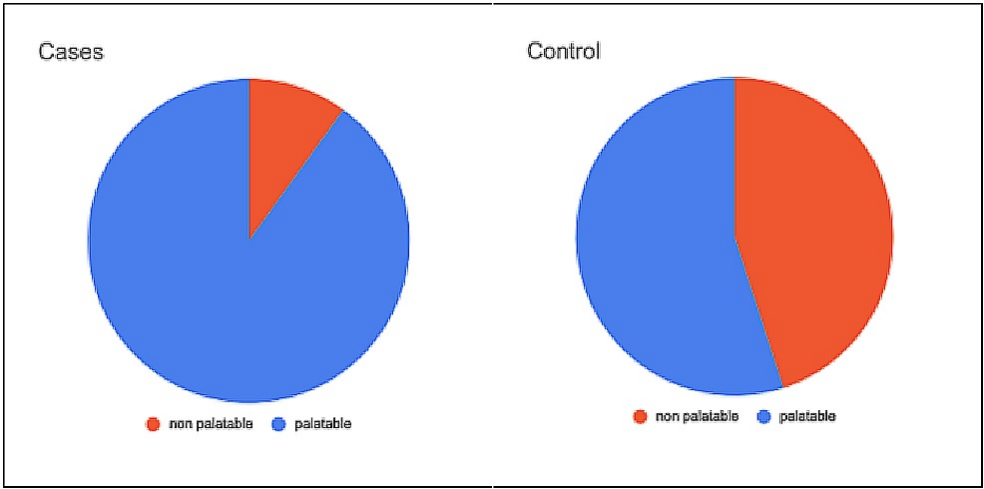
Pie chart comparing the palatability of lactulose and polyethylene glycol

**Figure 3:**
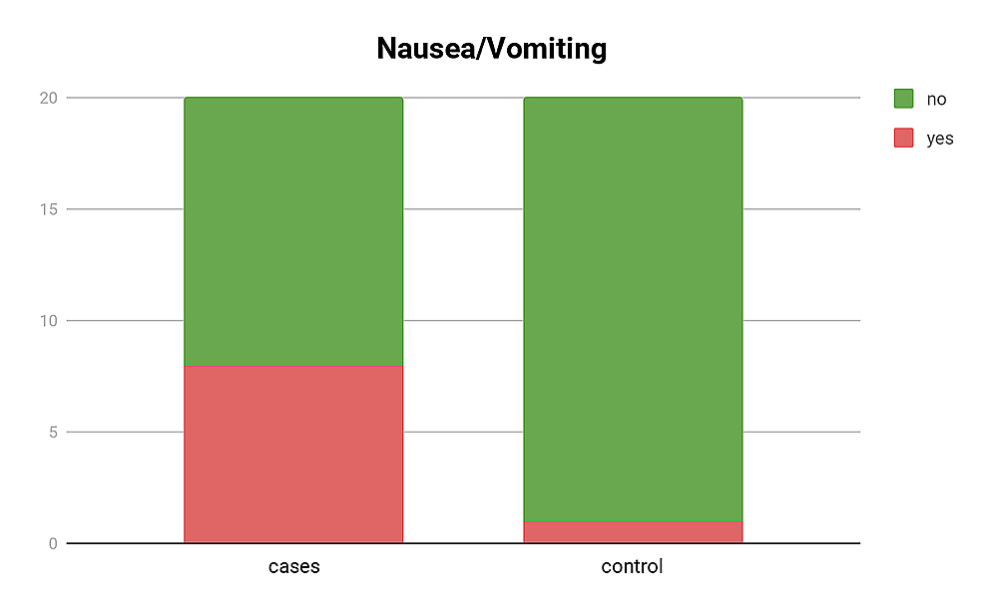
Graph comparing the occurrence of nausea/vomiting with lactulose and polyethylene glycol

**Figure 4:**
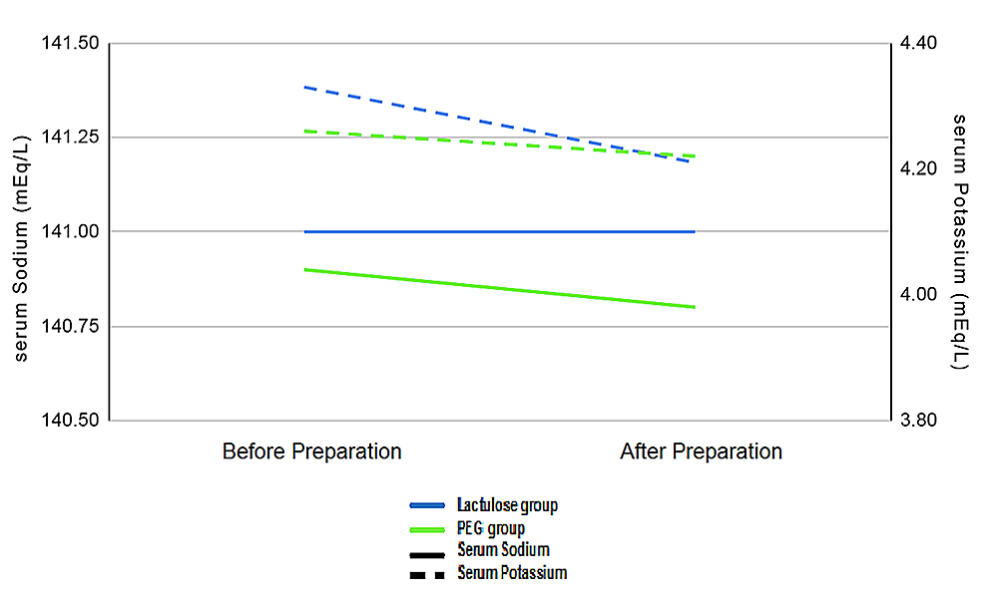
Graph comparing mean serum sodium and potassium levels before and after bowel preparation in both groups

**Figure 5:**
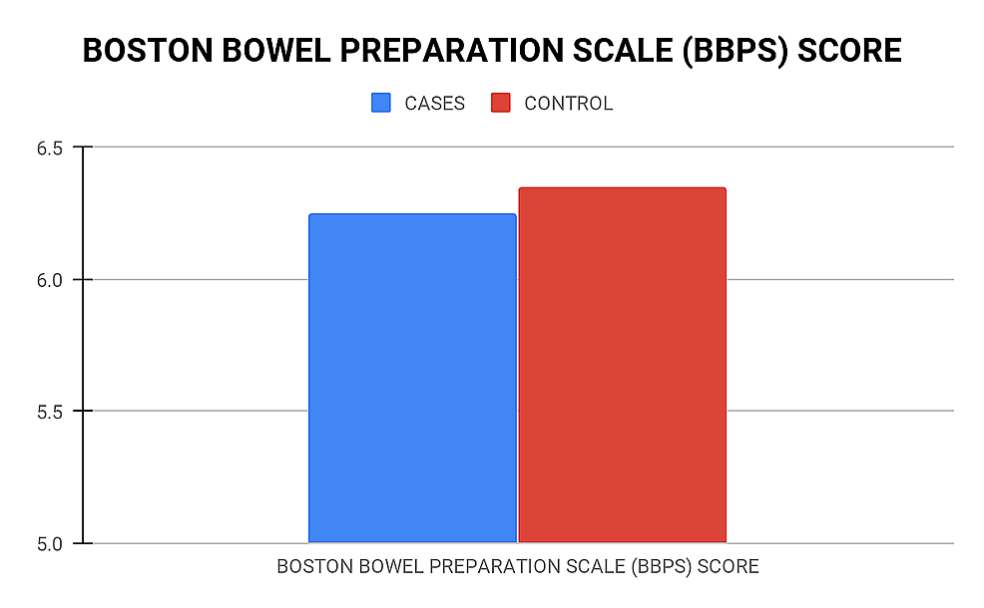
Graph comparing Boston Bowel Preparation Score in both the groups

A total of 40 patients were evaluated in our study. The mean age of the lactulose (Lac) group was 36.30 ± 8.986 years and the mean age of the PEG group was 34.35 ± 10.155 years (Table 2). The youngest patient was a 19-year-old male, whereas the oldest was 61 years. The male to female ratio was 19:1; there was a significantly higher incidence of bleeding per rectum in males (p-value <0.001). The most common cause of bleeding per rectum in our study was internal hemorrhoids (47.5%) while carcinoma of the rectum and carcinoma of the sigmoid was the least (7.5% and 2.5%, respectively). In the Lac group, 90% of patients found lactulose palatable and only 55% of patients found the PEG solution palatable, which was statistically significant (p-value=0.035) (Figure 2). Forty percent (40%) of the Lac group experienced nausea and vomiting as compared to only 5% in the PEG group, which is a significant difference (p-value=0.02) (Figure 3). Abdominal discomfort was experienced by 10% of patients in each of the two groups. There was no significant difference in serum sodium and potassium levels both before and after preparation in both the Lac and PEG groups (Figure 4). The mean BBPS scores of both the Lac and PEG groups showed no significant differences (6.25 ± 0.786 vs 6.35 ± 0.813) (Figure 5).

**Table 2:** Master table BBPS: Boston Bowel Preparation Scale; LGI: lower gastrointestinal; PEG: polyethylene glycol

Parameter	Lactulose (n =20)	PEG (n=20)	P-value
Age, Years	36.30 ± 8.986	34.35 ± 10.155	0.587
Sex (Male/Female)	19/1	19/1	
Cause of LGI Bleed			
Internal Hemorrhoids	9 (45%)	10 (50%)	0.819
Colitis	9 (45%)	8 (40%)	0.808
Ca Rectum	2 (10%)	1 (5%)	0.564
Ca Sigmoid Colon	0	1 (5%)	1.0
Palatability	18 (90%)	11(55%)	0.035
Nausea/Vomiting	8 (40%)	1 (5%)	0.02
Abdominal Discomfort	2 (10%)	2(10%)	1.00
Serum Sodium Levels			
before preparation	141 ± 5.46	140.9 ± 3.8	0.821
after preparation	141.0 ± 2.7	140.8 ± 3.1	0.838
Serum Potassium Levels			
before preparation	4.33 ± 0.07	4.26 ± 0.03	0.72
after preparation	4.21 ± 0.18	4.22 ± 0.17	0.923
Mean BBPS Score	6.25 ± 0.786	6.35 ± 0.813	0.59

## Discussion

Many bowel preparation agents are available today. These may be classified in multiple ways, including volume-administered (low volume versus high volume), osmolarity (isotonic versus hypo-osmotic versus hyper-osmotic), or main active ingredient (PEG), sodium picosulfate, and sodium phosphate (NaP). Insufficient cleaning can result in lower detection rates of incipient and advanced adenomas, flat lesions, and a higher rate of canceled procedures with increased costs, lengthier procedures, and a higher risk of complication [7-11]. The ideal preparation for colonoscopy should reliably empty the colon of all fecal material in a rapid fashion with no gross or histological alteration of the colonic mucosa. The preparation should not cause patient discomfort or a shift in fluids or electrolytes. The preparation should be safe, convenient, tolerable, and inexpensive [12-14].

In 2006, the American Society of Colon and Rectal Surgeons (ASCRS), American Society of Gastrointestinal Endoscopy (ASGE), and the Society of American Gastrointestinal and Endoscopic Surgeons (SAGES), state that a colonoscopy preparation should have the following properties: inexpensive, cleanse the bowels rapidly, and not a cause of significant patient discomfort or electrolyte imbalances [9]. Similar guidelines were published by the European Society of Gastrointestinal Endoscopy [2]. Three factors that are essential for a good bowel preparation are: safe, palatable, and efficacious. The use of oral bowel preparations may induce strong peristalsis, cramping, bloating, diarrhea, and others symptoms. Preparation intolerance is common and is usually associated with the volume of fluid consumed and the taste of the solution [15].

PEG is a non-absorbable electrolytic solution and does not induce mucus secretion of electrolytes or reduce the significant exchange of fluid in the colonic lumen. It is nontoxic but needs to be ingested in large quantities. Lactulose is a disaccharide. It is absorbed and undergoes bacterial action, which causes fermentation, acidifying the environment and causing acceleration of intestinal transit by stimulating motility [13-14]. In the colon, lactulose is broken down primarily to lactic acid and to small amounts of formic and acetic acids by the action of beta-galactosidase from colonic bacteria, which results in an increase in osmotic pressure and slight acidification of the colonic contents [13]. This causes an increase in stool water content and softens the stool and causes the acceleration of intestinal transit by stimulating motility [13]. Bowel preparation is generally safe for most patients but can be detrimental for patients with comorbidities if the wrong preparation is utilized on the wrong patient. In patients with congestive heart failure or chronic renal disease, the bowel preparation utilized should not result in massive fluid shifts or electrolyte absorption. Hence, polyethylene glycol electrolytic solution (PEG-ELS) is used in these patients and, generally, sodium phosphate preparations are avoided [16-18]. For these patients, especially the elderly with renal insufficiency or advanced liver disease with ascites, low-volume bowel preparation is ideal [5,19].

In our study, it was found that lactulose is more palatable than polyethylene glycol. Similar results were shown in other studies by Rendeli et al. and Sharara also [11,20]. But lactulose caused more nausea/vomiting than polyethylene glycol in the present study; these findings are also supported by Voskuijl et al. and Sanein in their studies [21-22]. However, in another study done by Chun-Xia Li, the administration of the lactulose oral solution, as compared with the PEG solution, was associated with a lower incidence of nausea (23.81% in the Lac group vs. 58.54% in the PEG group, p=0008) [23]. Both lactulose solution and polyethylene solution equally caused mild abdominal discomfort comparable to the results obtained by Attar et al. and Moraes in their respective studies [24-25] while there was no significant change in serum electrolytes in both the groups before and after bowel preparation, which is supported by the observations made by Shehata et al. [26].

In a study conducted by Menacho et al. in 2014, out of 200 patients, 100 patients were given lactulose and 100 patients were given polyethylene glycol as a cleaning agent for bowel preparation, and the Aronchick scale was used for assessing bowel preparation. There was no difference in the quality of bowel preparation in both groups [25]. Chun-Xia Li in their study of 176 patients observed that the Lac group had superior bowel cleansing compared to the PEG group (BBPS scores: 6 88 ± 1 78 vs 7 95±1 40, p-value = 0.001) [23]. In our study, the mean Boston Bowel Preparation Scale (BBPS) score in patients who received lactulose was 6.25 ± 0.786, and in those who received polyethylene glycol, it was 6.35 ± 0.813. The p-value was 0.590, which was statistically insignificant. The degree of cleanliness of the bowel preparation agent causing a BBPS score of <5 would likely be considered inadequate [6]. In both groups, BBPS was more than 6. Hence, we can conclude that both lactulose and polyethylene glycol are good cleansing agents for bowel preparation.

Limitations

Our study is not perfect, and it has the following limitations:

1. Sample size was less, as this was intended as a pilot study. Larger multicentric studies will be needed to substantiate the findings further.

2. The study only looks at the diagnostic aspect of colonoscopy (requires a comparison of tools of therapeutic interventions).

3. For the BBPS calculation, only the total score was taken, which was provided by the endoscopist. In our study, we did not give particular attention to compare each part of the large colon (further prospective studies about the efficacy of bowel cleansing agents may specifically look for the right/transverse/left colon individually).

## Conclusions

Lactulose is a good bowel cleansing agent, matching the quality of the polyethylene glycol solution that is routinely used. It also has a very favorable pharmacological profile. Lactulose is also just a fraction of the cost of the polyethylene glycol solution. Hence, it can be considered a cheaper alternative in resource-limited settings in low and middle-income countries.

## References

[1] (2020). American Cancer Society Guideline for Colorectal Cancer Screening. For people at average risk. https://www.cancer.org/cancer/colon-rectal-cancer/detection-diagnosis-staging/acs-recommendations.html.

[2] Hassan C, Bretthauer M, Kaminski MF (2013). Bowel preparation for colonoscopy: European Society of Gastrointestinal Endoscopy (ESGE) guideline. Endoscopy.

[3] Bechtold ML, Mir F, Puli SR, Nguyen DL (2016). Optimizing bowel preparation for colonoscopy: a guide to enhance quality of visualization. Ann Gastroenterol.

[4] Davis GR, Santa Ana CA, Morawski SG, Fordtran JS (1980). Development of a lavage solution associated with minimal water and electrolyte absorption or secretion. Gastroenterology.

[5] Marschall HU, Bartels F (2021). Life-threatening complications of nasogastric administration of polyethylene glycol-electrolyte solutions (Golytely) for bowel cleansing. Gastrointest Endosc.

[6] Lai EJ, Calderwood AH, Doros G, Fix OK, Jacobson BC (2009). The Boston Bowel Preparation Scale: a valid and reliable instrument for colonoscopy-oriented research. Gastrointest Endosc.

[7] Harewood GC, Sharma VK, de Garmo P (2003). Impact of colonoscopy preparation quality on detection of suspected colonic neoplasia. Gastrointest Endosc.

[8] Chokshi RV, Hovis CE, Hollander T, Early DS, Wang JS (2012). Prevalence of missed adenomas in patients with inadequate bowel preparation on screening colonoscopy. Gastrointest Endosc.

[9] Parra-Blanco A, Nicolas-Perez D, Gimeno-Garcia A, Grosso B, Jimenez A, Ortega J, Quintero E (2006). The timing of bowel preparation before colonoscopy determines the quality of cleansing, and is a significant factor contributing to the detection of flat lesions: a randomized study. World J Gastroenterol.

[10] Chiu HM, Lin JT, Lee YC, Liang JT, Shun CT, Wang HP, Wu MS (2011). Different bowel preparation schedule leads to different diagnostic yield of proximal and nonpolypoid colorectal neoplasm at screening colonoscopy in average-risk population. Dis Colon Rectum.

[11] Sharara AI, Abou Mrad RR (2013). The modern bowel preparation in colonoscopy. Gastroenterol Clin North Am.

[12] DiPalma JA, Brady CE (1989). Colon cleansing for diagnostic and surgical procedures: polyethylene glycol-electrolyte lavage solution. Am J Gastroenterol.

[13] Lee-Robichaud H, Thomas K, Morgan J, Nelson RL (2010). Lactulose versus polyethylene glycol for chronic constipation. Cochrane Database Syst Rev.

[14] Pulin Y, Zengjin L, Hong Z (2001). A survey of the current status and distribution of elderly constipation in China. Chin J Geriatr.

[15] Chatrenet P, Friocourt P, Ramain JP, Cherrier M, Maillard JB (1993). Colonoscopy in the elderly: a study of 200 cases. Eur J Med.

[16] Perkowska-Ptasińska A, Szewczyk K, Skuza A, Wasińska-Krawczyk A, Rydzewski A (2014). Phosphate nephropathy after administration of bowel purgative containing sodium phosphate - a case report. Pol J Pathol.

[17] Florentin M, Liamis G, Elisaf MS (2014). Colonoscopy preparation-induced disorders in renal function and electrolytes. World J Gastrointest Pharmacol Ther.

[18] Choi NK, Lee J, Chang Y (2014). Acute renal failure following oral sodium phosphate bowel preparation: a nationwide case-crossover study. Endoscopy.

[19] Day LW, Kwon A, Inadomi JM, Walter LC, Somsouk M (2011). Adverse events in older patients undergoing colonoscopy: a systematic review and meta-analysis. Gastrointest Endosc.

[20] Rendeli C, Ausili E, Tabacco F (2006). Polyethylene glycol 4000 vs. lactulose for the treatment of neurogenic constipation in myelomeningocele children: a randomized-controlled clinical trial. Aliment Pharmacol Ther.

[21] Voskuijl W, de Lorijn F, Verwijs W (2004). PEG 3350 (Transipeg) versus lactulose in the treatment of childhood functional constipation: a double blind, randomised, controlled, multicentre trial. Gut.

[22] Saneian H, Mostofizadeh N (2012). Comparing the efficacy of polyethylene glycol (PEG), magnesium hydroxide and lactulose in treatment of functional constipation in children. J Res Med Sci.

[23] Li CX, Guo Y, Zhu YJ, Zhu JR, Xiao QS, Chen DF, Lan CH (2019). Comparison of polyethylene glycol versus lactulose oral solution for bowel preparation prior to colonoscopy. Gastroenterol Res Pract.

[24] Attar A, Lémann M, Ferguson A (1999). Comparison of a low dose polyethylene glycol electrolyte solution with lactulose for treatment of chronic constipation. Gut.

[25] Menacho AM, Reimann A, Hirata LM, Ganzerella C, Ivano FH, Sugisawa R (2014). Double-blind prospective randomized study comparing polyethylene glycol to lactulose for bowel preparation in colonoscopy. Arq Bras Cir Dig.

[26] Shehata HH, Elfert AA, Abdin AA, Soliman SM, Elkhouly RA, Hawash NI, Soliman HH (2018). Randomized controlled trial of polyethylene glycol versus lactulose for the treatment of overt hepatic encephalopathy. Eur J Gastroenterol Hepatol.

